# “Will you be there for me?” Social support from family and friends during cold case sexual assault prosecutions

**DOI:** 10.1002/ajcp.70042

**Published:** 2025-12-26

**Authors:** Rebecca Campbell, Rachael Goodman‐Williams, Katie Gregory, McKenzie Javorka, Jasmine Engleton

**Affiliations:** ^1^ Department of Psychology Michigan State University East Lansing Michigan USA; ^2^ Department of Justice, Law and Criminology American University Washington District of Columbia USA; ^3^ The Rural Institute for Inclusive Communities University of Montana Missoula Montana USA; ^4^ SMU DataArts Dallas Texas USA

**Keywords:** disclosure, prosecution, rape kit, sexual assault, sexual assault kit, social support

## Abstract

If sexual assault survivors report the assault to the criminal legal system, they often need informal support from family and friends throughout the long and frequently retraumatizing process of investigation and prosecution. This study is part of a long‐term community‐based participatory action research project in a predominately Black/African American U.S. city that is facing extended delays in sexual assault prosecution because the police have not been routinely testing medical forensic evidence when sexual assaults were first reported. When police had this evidence tested years later, cases with DNA matches were re‐opened, and survivors were asked to re‐engage with the criminal legal system. We conducted qualitative interviews with *N* = 32 survivors who participated in these “cold case” prosecutions and explored how their family and friends supported them throughout this process. Most survivors (94%) disclosed to friends and family that their cases had been re‐opened, and 47% received beneficial emotional and tangible support during prosecution. The other half (53%) encountered indifference or blame from their family members and friends. We discuss how victim advocates can prepare survivors for the reactions they may receive from friends and family, and how community services can buffer loss of support.

## Highlights

- Sexual assault survivors need social support from family and friends during criminal prosecution.
- This study examined social support in “cold case” prosecutions that were delayed years.
- Approximately half of the survivors interviewed received help and support.
- The other half encountered indifference or blame from their family members and friends.
- Community‐based victim advocates may be able to buffer these negative reactions.

Sexual assault is a significant violent crime that causes devastating impacts on survivors' psychological wellbeing, physical health, and economic security (Fedina et al., 2020). To address these problems, survivors may turn to their communities to seek legal accountability and resources for their healing (see Campbell, 2024 for a review). However, survivors' help‐seeking experiences are often fraught and retraumatizing as they encounter victim‐blaming responses across all levels of their social ecologies (see Campbell et al., 2009; Stockman et al., 2023) for theory reviews). Survivors who choose to report the assault to the criminal legal system and participate in prosecution often encounter disrespectful, hurtful treatment from police and prosecutors, steeped in long‐standing victim‐blaming myths that women lie about being raped, that they provoke these acts, and that they should be silent and shamed (Edwards et al., 2011; Hlavka & Mulla, 2021). These adverse experiences with the criminal legal system are particularly common for Black women, who have historically been denied legal protection from sexual violence and continue to face racist and sexist victim blaming that evokes false narratives dating back to slavery about Black women being promiscuous and unrapable (Crenshaw, 1991; Edwards et al., 2011; Hlavka & Mulla, 2021; Kelley, 2023; McGuire, 2010; Richie & Eife, 2021).

Given these risks, survivors who do report to the police and seek prosecution often turn to informal sources of support, such as their family and friends, for respite and support as they navigate these formal systems (Kelley, 2023; Konradi, 2007). Social support from survivors' microsystems can be a critical resource for resisting and repairing the traumas victims experience in their interactions with exosystems and macrosystems (Ullman, 2024). For Black women, social support from family and friends has long been a key source of strength and collective healing from historical traumas and continued oppression and microaggressions (Bryant‐Davis et al., 2011; Catabay et al., 2019). However, reaching out to friends and family can also be risky, as victim‐blaming ideology permeates all levels of the social ecology (Campbell et al., 2009; Stockman et al., 2023). If individuals within survivors' microsystems ascribe to these prejudiced beliefs, they may replicate and reinforce—rather than repair—what victims encounter from societal macrosystems (Ullman, 2024). For Black women, seeking social support in the context of sexual assault can be particularly complex due to intracultural pressures to remain silent and not seek help or report to the police if doing so would reflect poorly on or harm a Black perpetrator and/or the Black community writ large (Gómez, 2023).

These tensions within and across ecological levels complicate the help‐seeking process: survivors may need support from their friends and family to help them through a fraught criminal legal process, yet there are relational risks to seeking that support. In this study, we delved into these tensions and explored what happens when survivors whose cases were being prosecuted by the criminal legal system reached out to family and friends for support. Our work is situated in a predominately Black/African American U.S. city, Detroit, Michigan, which provided an opportunity to elevate the experiences of Black women navigating informal social support during criminal prosecution. Furthermore, these were unusually complex legal cases. These survivors had reported their assaults to the police promptly and cooperated with legal personnel, but no arrests were made, and their cases went inactive for years, sometimes decades. Then, new DNA evidence came to light that prompted prosecutors to re‐open these “cold cases.” The survivors decided to once again engage with the criminal legal system and seek accountability (see Javorka & Campbell, 2025 regarding how they made these decisions). Our goal in this paper was to explore survivors' choices about whether to involve their family and friends for support as their cases were prosecuted. To set the stage for our exploration of survivors' social support experiences, we will begin with a review of prior research on survivors' informal support seeking, and existing work on how family and friends do or do not help when victims are navigating the criminal legal system. Then, we will explain the unusual circumstances that finally brought these cases to trial, and why informal social support is particularly critical for survivors in cold case prosecutions.

## The need for social support from family and friends

Sexual assault survivors are often hesitant to disclose the assault to people in their lives because they are unsure or fearful about how they will respond (Ullman, 2024). Overall, most victims (67%–80%) do disclose the assault to someone in their lives, typically family members and/or friends (Ullman, 2024). Sometimes survivors disclose because they are seeking emotional, informational, and/or tangible support regarding what they should do next (e.g., legal reporting, seeking health care), but others disclose without any intention of seeking formal help and just want to process what they have experienced (Ahrens et al., 2007; Ahrens et al., 2010).

How family and friends respond to these disclosures can be highly variable (see Ullman, 2024 for a review). Victims hope that people will respond with kindness, and most survivors receive at least one positive social reaction when they tell a family member and/or friend (Ullman, 2024). Survivors find it helpful when people listen without judgment, and when they offer tangible support (e.g., help with police, medical care, childcare, transportation), but allow them to make their own choices and control what options they pursue (Ahrens et al., 2007; Ahrens et al., 2010). Positive social support can help bolster survivors' psychological well‐being (Dworkin et al., 2019), especially for Black/African American sexual assault survivors (Bryant‐Davis et al., 2011; Catabay et al., 2019; Watson et al., 2012).

However, most survivors *also* receive negative social reactions from family and friends (Ullman, 2024). Relyea and Ullman (2015) interviewed a racially diverse sample of sexual assault survivors (45% were Black/African American, 35% White, 20% multiracial or other racial identities) and identified two common types of negative social reactions. First, “unsupportive acknowledgment” is when family and friends recognize that the victim was indeed assaulted, but they are distracted, distant, and/or egocentric in their interactions with victims and do not offer much support. Second, “turning against” is when family and friends actively blame survivors and/or accuse them of lying while withholding support and/or instigating additional stresses for victims. Black/African American women are more likely than White women to receive these negative social reactions when they disclose assaults to family and friends (Ullman, 2023). Gómez's (2023) cultural betrayal trauma theory offers an intersectional Black feminist explanation for why this occurs so frequently. Intra‐cultural ‘violent silencing’ pressures Black women to protect Black perpetrators from racially biased discrimination they may experience within the criminal legal system. When survivors *do* disclose and violate those norms, they may encounter blame, shame, coldness, and isolation from friends and family (Gómez, 2023; Kelley, 2023; Slatton & Richard, 2020). Perhaps unsurprisingly, these negative social reactions have adverse effects on survivors' mental health (Dworkin et al., 2019), especially for Black women (Long & Ullman, 2016; Washington, 2001).

## The need for social support during engagement with the criminal legal system

If survivors do disclose to family and friends, how those interactions unfold can shape whether survivors make a formal report to the police and pursue prosecution (Campbell et al., 2015; Lorenz et al., 2019; Patterson & Campbell, 2010). If those people seem hopeful that the legal system will take victims' cases seriously, survivors feel more encouraged to report (Campbell et al., 2015). However, for Black women survivors, there is generally less reason to be hopeful, as sexual assaults committed against Black women are chronically under‐prosecuted, regardless of the race/ethnicity of the offender (Shaw & Lee, 2019; Spohn, 2020). Despite these problematic patterns, Black women survivors will often report to the police—either with or without the support of their family and friends—if they are concerned that other women and girls could be harmed by their perpetrator and they want to prevent others from being assaulted (Kelley, 2023). Furthermore, if survivors experience the negative “turning against” pattern noted by Relyea and Ullman (2015), they may feel they *have* to report to the police to prove to the people in their lives that they truly were assaulted (Campbell et al., 2015; Gómez, 2023).

To date, there has been limited research on social support provided by family and friends after survivors file a police report. In a study of predominately White sexual assault survivors, Campbell et al. (2015) found that family and friends may disengage at the beginning of a criminal investigation as the difficulties of this process reveal themselves. For example, police may want to interview anyone survivors talked to and collect phone and text records of those communications (Patterson, 2011a). Family and friends have refused these requests and withdrawn support because they did not want to be personally involved in the investigation (Campbell et al., 2015). This disengagement is often interpreted by the police as an indicator that the people closest to the survivors do not find them credible, which increases the likelihood law enforcement personnel will close the case (Patterson, 2011b).

Likewise, few studies have explored what happens between survivors and their informal support networks as their cases progress to prosecution. Lorenz et al. (2019) interviewed a racially diverse sample of survivor‐support person‐matched dyads about social support provided/received throughout a legal case. Most survivors received positive emotional and tangible support from their family and friends, and this occurred regardless of whether their case was successfully prosecuted. In the interviews with the support providers, family and friends emphasized the need to stand with survivors, especially when they experienced disappointment and retraumatization from the criminal legal system. Informal support providers also felt frustrated about how survivors were treated by legal personnel, and victims were comforted that others were feeling their pain too. The importance of this unconditional support is underscored by the findings from Hlavka and Mulla's (2021) ethnographic study of sexual assault trials. Many cases in this project involved Black women survivors, who faced racialized sexist treatment in court, but many did not have in‐person social support from their family and friends.

## The need for social support in delayed cold case re‐engagement with the criminal legal system

As noted previously, many cases do not proceed past police investigation to prosecution (Spohn, 2020), and recently, a key reason why that gap persists has become clearer. For decades, police have advised sexual assault survivors to go to a hospital emergency department for a medical forensic exam and the collection of a sexual assault kit (SAK; also known as a “rape kit”) to preserve biological evidence, such as blood, semen, and saliva (see Campbell & Feeney, 2023 for a review). Victims consent to these exams with the understanding that the evidence will be tested for DNA, and the results will be used to investigate and prosecute their cases (Campbell & Feeney, 2023). However, police have not been routinely submitting rape kits for DNA testing, and instead, have been putting them in storage (Strom et al., 2021). Thus, critical evidence that could be moving more cases to prosecution has been systematically ignored. Currently, 96 U.S. jurisdictions, 33 of which are entire states, have large stockpiles of untested SAKs, which are commonly referred to as ‘rape kit backlogs’ (Sexual Assault Kit Initiative SAKI, 2025). Rape kit backlogs are more common in jurisdictions with high percentages of Black survivors and survivors from other marginalized and minoritized communities (Lovell & Fletcher, Sabo, et al., 2023).

Media coverage of these backlogs prompted public outcry, and in response, multiple federal agencies established programs to fund mass DNA testing of these older rape kits (Lovell & Langhinrichsen‐Rohling, 2023). This testing has yielded unexpectedly high rates of DNA matches to offenders and cross‐case DNA matches to suspected serial offenders (Campbell & Feeney, 2023). Based on that evidence, prosecutors across the country are beginning to conduct “victim notifications,” whereby police re‐contact victims to tell them their kits have finally been tested and the results can re‐open their legal cases (Campbell & Feeney, 2023). Victim notifications come as a tremendous shock to survivors and cause considerable disruption in their lives (Campbell, Gregory et al., 2023). Whether survivors share this unexpected news with family and friends and seek their support is not yet known. Given the complexities of cold case prosecutions, it is important to understand survivors' social support needs, particularly as these kinds of cases are becoming more common in the U.S. criminal legal system.

## The current study

Our goal in this study was to explore survivors' social support experiences with family and friends after they had received a cold case victim notification and decided to re‐engage with the criminal legal system to pursue prosecution. Given that these assaults happened years ago, survivors' options for social support are complex. Survivors may choose to *re‐disclose* to the same people they already told about the assault years ago, which raises questions about how those individuals might react now and whether they will be supportive. Survivors who received help and support years ago may wonder whether their family and friends will continue to stand by them. For those who experienced blame and betrayal years ago, survivors may feel hopeful that the new DNA evidence will vindicate them, and people in their lives will now be more supportive. Moreover, because victim notifications occur years after the assault and tend to up‐end survivors' daily lives, survivors may choose to make *new disclosures* to people in their current lives for the first time, such as new intimate partners and friends. Survivors may wonder whether these individuals will be supportive once they learn of these events from their past. Some survivors may choose to tell both “old” and “new” people in their lives.

As part of our 15‐year (thus far) community‐based participatory action research project with a predominately Black U.S. city that is resolving its backlog of untested SAKs (Campbell et al., 2021), our research team worked with community stakeholders to co‐design this study, co‐develop its research questions, select its methodology, and review draft results (see Campbell et al., 2022 for details about our CBPR approach). In this phase of our action research project, we interviewed *N* = 32 survivors who were among the first to participate in the prosecution of their cases after this city began mass testing its rape kit backlog. The current analysis focused on if and how their informal social support networks supported them throughout this process. We decided to utilize qualitative methods for this study because exploratory descriptive methods are appropriate for studying events that are novel and complex (Patton, 2015). In these interviews, we asked survivors who they told about their rape kit victim notifications and how their interactions with those individuals unfolded throughout the prosecution of their cases. With these data, we explored two research questions. Our first question was descriptive in nature: Did survivors disclose the rape kit victim notification to anyone in their lives, and if so, who did they reach out to? A key focus within this question was documenting whether survivors reached out to individuals who already knew about the assault and/or whether they disclosed to new people in their lives. Our second question was more in‐depth and sought to explicate and explain survivors' experiences: What support (if any) did those people provide, and was engagement with these individuals helpful to the survivors as they participated in the prosecution of their cases? A key focus within this question was comparing the support survivors received (or did not receive) in their initial disclosures years ago and their current support experiences during cold case prosecutions.

## METHODS

### Research design

We used a qualitative transcendental phenomenological research design that seeks to identify common features of a shared lived experience (Creswell & Poth, 2025), which in this study was survivors' experience of participating in a “cold case” sexual assault prosecution and the social support they received from their family and friends. Our aim was to develop descriptive depictions of this experience from the participants' point of view (Creswell & Poth, 2025). However, our positionalities as middle‐ and upper middle‐class, highly educated women (of different races, ethnicities, and gender and sexual identities) are markedly different than our research participants, most of whom were economically disenfranchised Black women. Throughout the project, our research team discussed these power dynamics, and we wrote reflexive memos to interrogate our positionalities and how they shaped our work (see Campbell, Goodman‐Williams et al., 2023 for our collective positionality statement).

### Sample

The target population for this study was sexual assault survivors who: (1) were 18 years or older at the time this study was conducted; (2) had been sexually assaulted in Detroit, Michigan; (3) had reported the assault to the police and had a SAK collected, but the police did not submit the kit for DNA testing at the time the crime was reported; (4) were notified that their SAK had been found in the city's backlog and sent for forensic testing, and that testing produced a DNA match; (5) participated in the re‐investigation of their case; and (6) participated in the criminal prosecution of their case. Over 20 months of recruitment, 112 survivors met these eligibility criteria.

We worked with this city's rape crisis center to develop a confidential, trauma‐informed recruitment protocol (Campbell et al., 2022). The agency's victim advocates had worked with all 112 eligible survivors during their cold case prosecutions (see Campbell, Gregory, Engleton, et al., 2025 for details about this community's multidisciplinary cold case team), and they agreed to reach out to survivors to explain the study and request their participation after all phases of their court cases were completed. The advocates successfully connected with 44 survivors (39% of eligible survivors): 33 whose cases ended in guilty pleas, 9 in trial convictions, and 2 in trial acquittals (case outcomes were known to the advocates because they had assisted the survivors throughout their re‐engagement). The advocates were unable to reach 68 survivors due to changed/unavailable contact information or no returned calls (61% of eligible survivors): 35 whose cases ended in guilty pleas, 19 in trial convictions, and 14 in trial acquittals. The advocates were less likely to be able to reach survivors whose cases ended in an acquittal relative to those whose cases ended in guilty plea or trial conviction: *χ* ^2^ [2, *N* = 112] = 7.85, *p* < .05. Of the 44 survivors who could be reached, *N* = 32 agreed to be interviewed (73% of eligible and reachable participants; 29% of all eligible cases). In the final sample of *N* = 32 survivors, *n* = 23 participants (72% of the interviewed sample) had their case adjudicated by a guilty plea, *n* = 8 (25%) by trial conviction, and *n* = 1 (3%) by trial acquittal. There were no differences between those who agreed and declined to be interviewed based on their legal case outcome: *χ* ^2^ [2, *N* = 44] = 1.86, *ns*).

All *N* = 32 participants identified as women; their average current age was 41.55 years old (SD = 8.74; range 25–60 years old) and their average age at the time of the assault was 23.06 years old (SD = 7.05, range 13–45 years old). Consistent with the demographics of this city, most participants identified as Black/African American (*n* = 28; 87.5%), three as White (9.1%), and one as multiracial (3.1%). All survivors experienced penetrative criminal sexual conduct, which would commonly be referred to as “rape”; however, in the research interviews, survivors used the terms “rape” and “sexual assault” interchangeably when referring to their experiences. Most survivors were sexually assaulted by a stranger (*n* = 23, 72%), and *n* = 9, 28% were assaulted by someone known to them (e.g., a friend, partner, or ex‐partner). Most survivors (*n* = 28 of *N* = 32) provided information about the gender and race/ethnicity of the assailant, and most were sexually assaulted by a Black/African American man (*n* = 27 of 28 who provided this information; *n* = 1 was assaulted by a White man). On average, these sexual assaults occurred 18.50 years ago (SD = 6.36, range 6 to 28 years ago). These research interviews were conducted on average 22.46 months since the survivors' cases were adjudicated (SD = 12.66 months, median = 19 months, range 5 months to 5.5 years, see Campbell et al., 2022 for more details).

### Procedures

The interviews were conducted in‐person at the city's rape crisis center or by phone, based on survivors' preferences (Campbell et al., 2022). The interviews lasted on average 80 min (SD = 29 min, range 36 to 171 min), and all participants consented to audio recording. Advocates were available to support participants if they became distressed during or after an interview, but none of the survivors requested this assistance. Participants were compensated $50 for their time and offered the agency's community resources brochure. The interviews were transcribed verbatim and checked for accuracy. All procedures were approved by the IRB of Michigan State University.

### Interview guide

We wrote a semi‐structured qualitative interview to guide the discussion with participants (Campbell et al., 2022). For the analyses presented in this manuscript, our primary data source was the interview sections discussing informal social support. All survivors were asked: “Who did you tell about being notified about the results of your kit?” Interviewers asked follow‐up questions about how each of those individuals reacted and what was or was not supportive to the survivor. As survivors described the prosecution of their cases, the interviewers continued to ask about the individuals to whom the survivors disclosed, whether they were involved in later stages of court, and if so, what support they did or did not provide to the survivor.

### Data analysis

Our research team (*n* = 5 interviewers/analysts) met regularly throughout data collection to review transcripts, discuss emergent ideas, identify issues to probe in future interviews, and assess emerging consistencies across survivors' narratives (see Braun & Clarke, 2021). After data collection was complete, two analysts used Atlas.ti (version 8) to group interview passages pertaining to the same topic (e.g., all text in which survivors described who they reached out to after their victim notifications was grouped together). The analysts also created simple inductive descriptive codes that summarized the basic content of a data passage (e.g., “called mother after notification,” “mother happy for her,” “survivor felt supported by mother's reaction”). One‐third of the transcripts were double‐coded until the coders reached complete consistency, and then they met regularly to review work and maintain consistency.

For the analysis presented in this paper, two other interviewers/analysts pulled all data passages and descriptive codes pertaining to disclosure and social support. To answer our first research question, we tallied how many survivors told someone about the victim notification, and if so, to whom they disclosed. We coded whether each disclosure was to someone who already knew about the assault (i.e., a re‐disclosure) or to someone previously unaware of the victims' assault history (i.e., a new disclosure).

To answer our second research question, we selected Braun and Clarke's (2022) reflexive approach to thematic analysis because it is well‐suited for identifying content themes and developing rich descriptions of those themes. Braun and Clarke (2022) outlined several core issues to be explicated prior to and throughout thematic analysis. First, for the scope of this analysis, the broader data corpus included all interview excerpts about victim notification, re‐investigation, and prosecution, and the data set for this analysis was excerpts in which survivors described their interactions with family and friends throughout that process. Second, for our guiding epistemological framework, we adopted a pragmatic, realist approach in which participants' words were assumed to reflect their meaning and experiences. Third, for our analytic approach, we chose inductive, bottom‐up coding to identify content themes that described common and novel experiences across participants.

With these foundational decisions established, we then followed Braun and Clarke's (2006, 2022) six phases of thematic analysis. We *familiarized ourselves with the data* throughout interviewing by group debriefing and reviewing transcripts. We *generated initial thematic codes* by assigning short codes to text segments to capture the nature of the support provided (or withheld) and the impact of that interaction (e.g., “friend listened to survivor process emotion, which made her feel heard,” “father accompanied survivor to court, which made her feel safer”). We then *searched for themes* by sorting and condensing the initial thematic codes into higher‐order themes. Next, we *reviewed and evaluated the adequacy* of the themes by assembling illustrative quote excerpts for each theme. For example, we considered whether there were two distinct themes regarding survivors' positive social support experiences (emotional vs. instrumental support provided), but when we reviewed the assembled evidence, it was clear that the type of support was not a focus for survivors as much as the impact of that support; hence, we retained one merged theme. With this organization of the findings settled, we *defined and named the themes* and finalized the selection of quote excerpts to ensure diverse representation of survivors. Finally, we *wrote this textual report* of our findings, which included bringing details about the survivors' assault experiences into the narrative for context.

## RESULTS

### RQ1: Survivors' disclosures to family and friends after cold case SAK victim notifications

Nearly all survivors we interviewed (*n* = 30 of *N* = 32, 94%) told someone in their lives that their rape kit had finally been tested and that their sexual assault criminal case would be re‐opened. All 30 survivors made at least one disclosure to someone who already knew about the assault (i.e., a re‐disclosure). The most common people survivors re‐disclosed to were their mothers (*n* = 11), followed by a sibling (*n* = 9), a friend (*n* = 7), other family members (e.g., father, aunt, grandparent, cousin; *n* = 7), and/or current/former intimate partners (*n* = 2).^1^ Eleven of these 30 survivors also made new disclosures to people who were previously unaware of the assault: friends (*n* = 2), their children (*n* = 7), and/or current intimate partners (*n* = 4).^2^ The seven survivors who made new disclosures to their children (who were now adolescents or adults) did not engage their kids as a source of support during legal re‐engagement (i.e., children were not actors in survivors' narratives pertaining to RQ2).

### RQ2: Survivors' support experiences with family and friends during cold case prosecutions

We examined the narratives from all *N* = 32 survivors we interviewed to explore if and how family and friends supported them throughout the re‐investigation and prosecution of their cases. We included the two survivors who did not tell anyone about the notification in these analyses because their reasons for *not* disclosing helped illustrate the dynamics of social support in cold case sexual assaults. We identified three themes in survivors' narratives about the social support they received—or did not receive—throughout their cold case prosecution. The first theme reflected how survivors' families and friends provided emotional and instrumental support and truly stood with them throughout their entire journey to justice. The second theme captured how some of the survivors' friends and family did not provide support, and their cold, detached response was upsetting and isolating. The third theme revealed how some survivors were actively betrayed and harmed by family and friends when they had turned to them for support. There was no overlap among the themes, such that those who described supportive interactions (Theme 1) did not mention negative experiences (Themes 2 & 3). In our analysis, we did not presuppose or impose mutual exclusivity between themes; rather, we focused on identifying patterns in survivors' social support experiences. However, our results showed that themes were in fact mutually exclusive; survivors who received care and support from family members did not mention cold or hurtful responses from those individuals (and vice versa).

#### Supported throughout: “They stood with me” (Theme 1)

Fourteen survivors discussed how their family and friends stood with them throughout the long and difficult process of being raped, receiving little to no help from the police, and then being notified years later that their cases were being re‐investigated and prosecuted. As one survivor said about her family and friends, “they were there before… still here now… they were always with me” (Participant S14). After the victim notification, many survivors struggled with traumatic flashbacks, which grew more intense as they prepared for court and testified in preliminary hearings. It was helpful for survivors to be able to talk to family and friends who already knew what they had been through and were still there for them as they processed the reactivation of traumatic memories. For example, one survivor we interviewed was assaulted 25 years ago when she was 21 years old. She had been carjacked at gunpoint and raped by a stranger, and after her victim notification, she told “everyone in my family, my best friend… they were very supportive” (Participant S19). As her legal case dragged on for months and she experienced more intense trauma symptoms, she leaned on her best friend, “I talked to her a lot during that time” (Participant S19). Another survivor who had been raped by a masked stranger 22 years ago when she was 19 years old described how her family and best friend rallied around her throughout the entire process:

[My sisters were] very supportive through the process… my best friend was my biggest support. When I felt down with anything I cried about, she would comfort me, she stands by me…from the beginning to the end, they stayed with me and they supported me.

(Participant S27)

This survivor was one of the four we interviewed who made a new disclosure to her current husband, and she described how he joined with her family and friends, who already knew about the assault, and offered unconditional support:

I still have nightmares, I still have panic attacks … my husband, no judgment, none …I didn't want him to look at me no different, and he has never…he was my biggest support when I went throughout my rape trial, he was there, my support, my rock.

(Participant S27)

This steadfast support was critical because the reality of facing the assailant in court and giving testimony in public was frightening for survivors. One survivor who had been raped 22 years ago when she was 31 years old was terrified to see her former friend, who “turned on her” one night and knocked her out, and dragged her into his garage to rape her. She described how she tried to cope with that fear:

The night before [court], I got so drunk and [the next morning] my friends [said], “You gotta go,” and I'm like, “I ain't going nowhere.” They got me coffee, [they said] “you gotta sober up because you gotta do this”… one of my girlfriends went with me, she was by my side.

(Participant S12)

In this case, the survivors' friends did not judge her for coping with her stress and fear by getting drunk. They knew from prior disclosures and conversations that she truly wanted to participate in the prosecution of her case and ensure that the perpetrator could not harm other people, so when she faltered, they literally and figuratively “propped [her] up” for court.

Survivors also described how family and friends had also been carrying the weight of this trauma over the years and how they shared their joy and relief. For example, one survivor who was carjacked and raped by a stranger 20 years ago when she was 19 called her best friend right after the notification, “I said to her, ‘You won't believe what happened,’ and she's like, ‘holy [expletive] after all these years?…that's awesome.’ She was very supportive” (Participant S33). Similarly, a rape survivor who had been abducted while walking home from work 17 years ago, when she was 21 years old, described how her family had been holding that pain with her and were happy she had another chance at justice:

The first person I called [after the VN] was my aunt, “Girl can you believe this.” I didn't even get the chance to finish what I was saying because my aunt finished my sentence. She said, “they got the person that raped you.” How did she know that was what I was going to say? But she hit it right on the nose…I told the other family too, and everyone was relieved and happy.

(Participant S30)

This survivor's family offered to stand with her throughout her court hearings: “[my family said] they would be there for me if I needed to go to court if I wanted somebody there with me…My aunt really wanted to go [to help me] … and she did” (Participant S30).

Some of the survivors we interviewed highlighted how it was especially meaningful to them that the men in their lives—their fathers, brothers, husbands, and even ex‐partners—stood with them throughout this entire journey and appeared with them in court. Their presence helped mitigate the stress of testifying in court and answering cross‐examination questions from defense attorneys, which often attacked victims' character and credibility. Although they did not want the men in their lives to hear these things, survivors felt validated that their male support providers came to court and let their presence signal that they believed the survivor. For example, one woman we interviewed had been raped over 10 years ago when she was 22 years old by an acquaintance who drugged her at a social event, and gang raped her with his friends. Years later, in court, she was angry that the defense attorneys implied that she consented to these acts. She was grateful that, “My dad was there at the court with me, he was there so I wasn't by myself. I had a family member that was there…and my dad believed me” (Participant S24). Likewise, another survivor who had been abducted and raped at gunpoint had her credibility attacked in court because she was a former sex worker. She described how her ex‐boyfriend stood with her at court: “[my ex‐boyfriend] was around when it happened [the night she was raped]. He went to court with me several times …he came and stood with me” (Participant S4). Her ex‐partner did not have to come to court to support her, but he did, and his continual support from the night of the attack to the day she testified in court years later was typical of the narratives within this theme.

#### Disregarded then and now: “Keep that to yourself” (Theme 2)

In contrast to Theme 1, *n* = 12 survivors discussed how their family and friends were cold and distant after they told them about their victim notifications. All these survivors had disclosed to these same individuals at the time of the assault, and their responses at the time reflected some sympathy, but overall, more apathy. Their family and friends acknowledged that the survivors had indeed been assaulted (in contrast to Theme 3 below), but they also indicated they were not available (emotionally or tangibly) to help, so victims would need to work through their trauma on their own. Years later, these survivors reached back out to the same individuals, hoping that the prosecutor's decisions to charge the perpetrators might help their family and friends appreciate the seriousness of these cases, but they were again met with indifference. For example, a survivor who was robbed and raped by multiple assailants 24 years ago told her mother and sister about her rape kit test results and her case being re‐opened; she characterized their response as:

They were just a little bit, “Oh yay, then they'll be brought to justice.” But still like, “Keep that to yourself.” It was like, ‘Oh, you're sad. Ho‐hum. I was thinking about short ribs for dinner.” That type of thing.

(Participant S25)

Similarly, another survivor who had been raped 25 years ago had told her mom at the time that she had been waiting at a bus stop to go to work when a stranger accosted her a gunpoint and forced her behind a nearby building where he sexually assaulted her. She had hoped her mother would support her years later when the case went to court, but she did not: “I figured I could tell her…I mean she had it to where we could talk about what we felt, but [there's] a limit… like she didn't want to hear it…it wasn't really supportive to me” (Participant S20). These survivors received acknowledgment from family and friends, but they wanted both acknowledgment *and* active support.

Some survivors described how family and friends were self‐centered, expressing more concern about how the assault and subsequent court hearings could negatively impact them (rather than the victim). For example, one survivor we interviewed had been abducted at gunpoint at a bus stop when she was 20 years old; the stranger raped her and beat her so severely that she sustained serious injuries. When she came home from the hospital emergency room after the attack, her mother and sister were more focused on how their own lives might be inconvenienced because of the survivor's injuries:

My mom was like, “Well, that doesn't mean you're not going to go to work tomorrow is it? You need to pick up my food.” My sister was like, “Gee, I've never known anybody that's been assaulted. That's like dirty me up” … I couldn't really get them to understand, I'm really not okay… they were like, if you let it be a problem for you, then I guess that's you, but I can't really go through that with you.

(Participant S26)

Years later, this survivor decided to tell her mother and sister about her victim notification and upcoming court dates, hoping that they might finally recognize the gravity of the situation and offer help. Unfortunately, “it was the same thing later” (Participant S26). This survivor received an equally cold reaction from her grandmother when her court date interfered with her ability to help her grandmother: “I told [my grandmother] about the case …She didn't even care. She looked at me and said, “be careful” and she didn't care…I almost died that day. She's just cold hearted” (Participant S26). Similarly, another survivor we interviewed had been abducted and raped 13 years ago when she was 19 years old, and although her mother did not offer much help at the time of the assault, she decided to tell her about the case being re‐opened because she hoped her mother would now be more supportive. However, her mother was preoccupied with the possibility that she could be called as a witness, and she wanted no part of her daughter's court case:

I called my mom [to tell her about the kit testing], and she was like, “Your business is your business. I don't want to know, just don't have them call me.” That was her response…I had no one there for me… I had no support of my own outside of the advocates.

(Participant S1)

Indeed, all 12 of these survivors went to their court hearings and trials without any family or friends with them for support, as one summarized: “I went through all of it, to me, by myself without family or anything, standing there all alone” (Participant S10). These survivors did not want to be alone, and they asked their mothers, siblings, friends, and/or family members to come with them to court, and one by one, each one refused. For instance, one survivor we interviewed was raped 24 years ago when she was 18 years old by a stranger with a gun after she got off the bus. She was relieved her case was finally going to be prosecuted and wanted someone to be with her as she faced the assailant:

I didn't feel too much like she [my sister] wanted to be there. I had to ask her [if she would go to court with me] it seemed like she didn't want anybody to know and she didn't go …I told my father, but he couldn't come…. I talked to my father's brother's wife [she declined]… I just felt like I'm in here alone without some family.

(Participant S29)

The lack of family presence and support had additional negative consequences for survivors. The perpetrators often had their family and friends in court to support them, and these survivors had to stand alone. Victims worried about being harassed and/or threatened by the perpetrator's supporters, and with no one from their own families there, they felt especially vulnerable. For example, as one survivor who was raped by a stranger 17 years ago when she was 21 years old explained:

My family, they didn't even care. My mother didn't even go with me [to court], and I asked her, can she? She was like, no…She just didn't want to bother with that, I had no one… he had plenty of family there, like his dad, and it was like older folks, and a young person or two in there. Maybe a sister or a mother. I was straight up scared.

(Participant S15)

These reactions—coldness and refusals to help—were precisely why one of the two survivors who did not tell anyone about the victim notification decided to “do it alone” (Participant S3). This survivor had been raped 10 years ago when she was 24 years old by a friend who tricked her into coming with him to an abandoned house, where he sexually assaulted her. When she told family members at the time of the assault, she experienced indifference from one person (“I didn't feel like I was really supported [by her]”), and self‐centeredness from another (“she made it all about her”). This victim was worried she might receive the same response again, so she made a self‐protective choice to keep this information to herself: “There wasn't really anyone else to talk to about it anymore…as a person, I've learnt how to deal with a lot of the bad things that happen in life by myself” (Participant S3).

#### Betrayed again: “They said I was lying” (Theme 3)

Theme 3 was a small, but distinctive set of narratives from four survivors who recounted how they felt betrayed, blamed, and harmed by members of their family and friends when they disclosed the rape years ago, and, unfortunately, they experienced the same treatment when told them about their victim notifications. Unlike the narratives in Theme 2, these survivors did not receive acknowledgment that they had indeed been raped; instead, their family members and friends accused them of lying about the assault, which was profoundly hurtful for these survivors. Three of the four survivors who were accused of lying about the assault had been assaulted by someone they knew—and their families and friends knew as well. It was surprising and shocking to these survivors that the people closest to them did not believe them and sided with their perpetrators. For example, one of these survivors had been raped 11 years ago when she was 14 years old. She had been hanging out with her older sister, her sister's boyfriend, and his friends one night and was pressured to accept a drink that turned out to spiked with drugs and she passed out quickly. When she regained consciousness, she realized she was being raped by her sister's boyfriend. She described her family's reaction at the time as:

My family's just like, nobody supports anyone…that's how it was even after that happened [the assault years ago]. It was kind of like, this happened and never talk about it again… my mom never did anything about it…she didn't believe me … they thought I cheated with my sister's boyfriend … and made it [the allegation of rape] up.

(Participant S11)

Then years later, when her rape kit tested and case prosecuted, her family reacted the same way:

They were like, oh wow, really?…And then it was just like, never talked about it ever again…So, again to me it felt like even she didn't believe me…even now, she still didn't believe me… she was all about my sister … and him [the perpetrator]…yeah, I'm on my own.

(Participant S11)

Survivors hoped the DNA evidence would finally put to rest their accusations of lying. For example, one survivor described how her family members actively disputed her account and widely shared their belief to others that the survivor was lying. This woman had been raped by a family friend 22 years ago when she was 18 years old. Prior to the assault, the victim's family encouraged her to begin a relationship with this man, and they were starting to spend more time together when one night he broke into their home and strangled and raped her. When the victim told her mom what happened, “She [the mother] told everybody I didn't get raped, but at this point, why would I lie? … She was telling people also in the neighborhood that it wasn't a rape” (Participant S22). Years later, her family still didn't believe her when she told them about the DNA test results confirming the identity of the offender and the prosecutors deciding to issue criminal charges. When the case was moving forward to trial, the case detectives let the survivor know that both her mother and her sister refused to testify on her behalf:

My sister was supposed to testify [but she refused] … The detective said [my sister] said, “I don't want to [expletive] testify… I'm a felon and this can mess with me.” And I asked the detective, “By her having a felony, her testifying can mess her up?” The detective was like, “No.” [My sister] just didn't want to do it, so I just did it myself … I was really hurt.

(Participant S22)

Despite the lack of corroborating witness testimony, the prosecutor was able to secure a guilty plea bargain from the offender based on the rape kit evidence and the survivor's testimony. As the victim recounted with sadness, “I just did it myself.”

Similarly, another survivor hoped her family would finally believe her that she had been raped 24 years ago when she was 27 years old. She had been hanging out at a friend's house, and when she tried to leave for the evening, two men blocked her exit, restrained her, and gang raped her. When she told family and friends—including the friend whose house this happened at—they didn't believe her. Because the police did not arrest the offenders, who had been named and identified by the victim in the police report, the survivors' social network interpreted the inactions of the police as proof that she was lying:

They thought I was lying. Because they figured, well, if you went and did a rape kit, why didn't they do anything about it? The system made me look like I was lying and made up a whole story…no one believed me… they made for a liar.

(Participant S9)

Years later, when the rape kit was tested, and the prosecutors issued charges against the offenders, the survivor said, “I finally felt relieved. That I was going to get closure, and prove to everybody that said I was lying, that I was not lying.” However, people still chose not to believe her: “I actually called the girl, whose house it happened at. She was angry with me. As if I did something wrong. She stopped being my friend, again… she still thought I made it up. (Participant S9)

These kinds of betrayals were the reason why the other survivor, who did not tell anyone about her victim notification decided to keep quiet. This woman had been raped 15 years ago when she was 19 years old, and like many of the women we interviewed in this study, she had been abducted by a stranger and raped repeatedly. When she told her mother what happened, she didn't believe her:

She thought I was lying then. Her boyfriend was all in her ear and said I was lying, I just ran off with somebody. Everybody dismissed it like nothing happened … and then the police treated me like it never happened. So it kind of felt like it never happened.

(Participant S7)

Because the police did not take any action in her case, the survivor's family and friends interpreted that inaction as more proof that she was lying. When DNA testing identified the offender years later, and the case was re‐opened, we asked her whether she shared this information with others, to which she replied, “No… why would I? I learned then they didn't believe me” (Participant S7).

## DISCUSSION

When survivors reach out for help after being sexually assaulted, they need support, caring, and resources from both informal and formal help systems (Ullman, 2024). In this study, we had an opportunity to explore if and how survivors' friends and family provided social support while victims were navigating exceptionally complex legal prosecutions. Our project is part of a long‐standing, multi‐year collaboration with one of many U.S. cities that has failed rape survivors by leaving critical medical forensic evidence untouched and untested for decades. This city discovered its rape kit backlog in 2009 and has since made significant strides to test all its kits and to begin to move cases forward for prosecution (Campbell et al., 2021). This progress is particularly meaningful as most of the survivors in this backlog are Black women, who were disbelieved and silenced by the police when they reported the assault years ago. However, this “second chance at justice” was not easy, and these survivors faced significant challenges during their victim notifications and throughout their court cases (Campbell et al., 2023; Campbell, Gregory, Goodman‐Williams, et al., 2025). They wanted support from their family and friends, and some (47%) received the support they needed. However, the other half (53%) were met with indifference or blame.

The results of this project are consistent with prior studies that have found survivors receive mixed reactions from family and friends, and for many, social support is elusive. Gómez's (2023) cultural betrayal trauma theory explains why this is common for Black women, who face intra‐cultural pressures to not report Black offenders to the police due to long‐standing racial discrimination by the criminal legal system (Crenshaw, 1991; Richie & Eife, 2021). When Black women survivors do report to the police—which they are more likely to do if they are concerned the perpetrator will harm others—their informal support networks may not support them. In this study, we extended this literature by examining social support *after* survivors decided to engage with the criminal legal system and to document how support unfolds over the long process of prosecution. Furthermore, because these cases had been delayed for years, we could examine consistency in social support from family and friends over time.

Nearly all survivors re‐disclosed to family and friends who already knew about the assault from prior disclosures years ago. How those individuals reacted to learning that the victims' cases being re‐opened was highly consistent with how they had responded to the survivors' initial disclosures. Many survivors received kindness and care, and this consistent support over time gave survivors tremendous relief. Our results replicate Lorenz et al.'s (2019) findings that survivors appreciated when others “carried” their feelings too and shared their experiences of hope as well as sadness. Interestingly, only six survivors made new disclosures to their current intimate partners and new friends, and all those new disclosures unfolded positively. These individuals accepted this information without blaming the survivors and joined with others in their social support network to help victims process their trauma and fear. Survivors described how helpful it was that their friends and family (new and old) stood with them in court to face their perpetrators. Given deep intra‐cultural norms about protecting Black men from discrimination, it was significant and poignant for survivors when the men in their lives (e.g., fathers, partners, ex‐partners) came with them to court to signal their support with their physical presence.

However, as has also been documented in the literature, we found that many survivors did not receive help and support from their family and friends—either at the time of assault or when their cases were re‐opened. Unfortunately, this consistency over time was intensely hurtful to survivors, who had hoped that the reasons why their cases were re‐opened—DNA evidence, prosecutor engagement, community mobilization about the rape kit backlog—might change how people treated them. Our results replicate Relyea and Ullman's (2015) findings that some survivors received unsupportive acknowledgment: family and friends conveyed a general belief that the survivors had been harmed, but did not offer emotional warmth or tangible support. We did not conduct dyadic interviews, as did Lorenz et al. (2019), and thus we do not know why people responded as they did, but other research provides some insight into why this may be happening. Campbell et al. (2015) found that family and friends did not want their own lives impacted by the victims' court cases, and in this study, survivors discussed this same problem. Again, in the city in which this study was conducted, which has faced decades of over‐policing and racial discrimination (Campbell et al., 2021), involvement with the criminal legal system is not without risk. Survivors had hoped their family and friends would make a choice to stand with them, but they encountered more disappointment and hurt when they did not.

We also observed a less common but still distinctive pattern of family and friends turning against survivors, which also replicates Relyea and Ullman's (2015) findings. Four women we interviewed described how people in their informal support networks accused them of lying at the time of the assault, and despite new DNA evidence and new court hearings, they still did not support the survivors. In three of these four cases, the assailants were someone known to the victim—and to her family. The assailants were friends and/or dating partners of family members, and they had engendered the family's trust and regard. When survivors accused these men of rape, their behavior was called into question, as was the judgment of the family members who held them in regard. In the end, the families chose to believe the perpetrators, not the survivors. As Gómez (2023) noted, this betrayal meant that the pain of the assault itself and everything thereafter (e.g., prosecution) was “hers (alone) to bear” (p. 59). That said, we note that our study was not conceptualized as a formal evaluation of cultural betrayal trauma theory, and we did not directly ask survivors about the violent silencing they experienced within their social networks. We highlight this omission to emphasize how future research on sexual assault disclosure and help‐seeking among Black women can be informed by Gómez's (2023) work to bring more cultural nuance to the study of social reactions and victims' involvement with the criminal legal system.

There are additional limitations of this study that merit reflection. First, this study's sample under‐represents survivors whose cases ended in trial acquittals. Our victim advocacy community partners cautioned us that victims whose perpetrators were found not guilty might be reluctant to participate in our study. If those survivors experienced indifference and/or betrayal from their family and friends at the time of the assault and during re‐engagement, it stands to reason that those reactions may have intensified if the case outcome was an acquittal. Second, consistent with a qualitative approach, we asked open‐ended questions to invite participants' reflections about their disclosure and social support experiences. This method yielded rich, detailed data, but established quantitative measures, such as the Social Reactions Questionnaire (SRQ; Ullman, 2024) could be used in future research to create directed prompts to be asked of all participants. We encourage other research teams to consider how to leverage the content of existing quantitative measures in their qualitative studies.

With these limitations noted, our results may be useful for practitioners working with sexual assault survivors in cold case prosecutions. Survivors need both emotional and tangible support, given how re‐opening these legal cases up‐ended their lives, which underscores the importance of trauma‐informed community services to help those who do not have informal support. This community created a multi‐disciplinary, trauma‐informed response team, so all survivors in this study had the assistance of confidential community‐based advocates throughout their cases, which they said was tremendously helpful (Campbell et al., 2024). However, survivors noted that they were typically connected with their advocates *after* the victim notifications, and it was in that gap of time that many disclosed to family and friends for support. Survivors emphasized that advocates should be present for the notifications, which would also give them an opportunity to prepare and strategize with survivors about if and how they engage their family and friends. Our results also underscore that if survivors seek counseling services during or after their court cases are resolved, clinicians may need to assist them with healing from not only the trauma of the rape and the court case, but also the betrayal and harm survivors encountered from their families. Prior research has found that women of Color are less like to reach out to rape crisis centers/victim services agencies for advocacy and counseling services (Ullman, 2024). As part of this city's response to its rape kit backlog, there have been sustained efforts to increase funding and community visibility of its victim service agency and its culturally specific programming (Ayeni, 2022; Campbell et al., 2021). Our results suggest that it would be helpful for these programs to develop family‐focused healing resources and to promote broader community dialog about intra‐cultural norms that can silence Black women (see Ayeni, 2022 as an example).

Sexual assault survivors deserve help and support as they seek the accountability and resources they need to heal from violence. The U.S. criminal legal system is adversarial, discriminatory, and punitive (Crenshaw, 1991; Richie & Eife, 2021), and it is risky for members of marginalized and minorities communities to trust such a system to report a violent crime. Sexual assault survivors face substantial challenges throughout the process of investigation and prosecution, particularly if they are Black women, as their cases are less likely to be investigated, their rape kits are less likely to be tested, and their cases are less likely to be prosecuted (Shaw & Lee, 2019; Spohn, 2020). Despite these patterns, many Black women survivors do report their assaults to the police to try to protect public safety, and they often need help from their informal support networks to withstand this arduous, victim‐blaming system. Sexual assault survivors do not want to have to choose between their families and friends and the one option our society offers for accountability (Gómez, 2023). Our study demonstrates that when survivors *do* have the support of their family and friends, their positive engagement meaningfully buffers the stresses and traumas they experience during prosecution. Strengthening informal support networks between survivors and their family and friends is critical for promoting survivors' well‐being.

## CONFLICT OF INTEREST STATEMENT

The authors declare no conflicts of interest.

## Data Availability

Project materials and data are available at the National Archive of Criminal Justice Data: https://doi.org/10.3886/ICPSR38921.v1.

## Footnotes

1 These tallies do not sum to 30 because most survivors told multiple family members and friends.

2 These tallies do not sum to 11 because most survivors who made new disclosures told multiple individuals. One survivor also made a new disclosure to a formal support provider (her current therapist).

## References

[ajcp70042-bib-0001] Ahrens, C. E. , Campbell, R. , Ternier‐Thames, N. K. , Wasco, S. M. , & Sefl, T. (2007). Deciding whom to tell: Expectations and outcomes of rape survivors' first disclosures. Psychology of Women Quarterly, 31(1), 38–49. 10.1111/j.1471-6402.2007.00329.x

[ajcp70042-bib-0002] Ahrens, C. E. , Stansell, J. , & Jennings, A. (2010). To tell or not to tell: The impact of disclosure on sexual assault survivors; recovery. Violence and Victims, 25(5), 631–648. 10.1891/0886-6708.25.5.631 21061869

[ajcp70042-bib-0003] Ayeni, O. O. (2022). Examination of a culturally specific sexual assault intervention for African American female survivors. Journal of Aggression, Maltreatment & Trauma, 31(8), 996–1013. 10.1080/10926771.2022.2089861

[ajcp70042-bib-0004] Braun, V. , & Clarke, V. (2021). To saturate or not to saturate? Questioning data saturation as a useful concept for thematic analysis and sample‐size rationales. Qualitative Research in Sport, Exercise and Health, 13(2), 201–216. 10.1080/2159676X.2019.1704846

[ajcp70042-bib-0005] Braun, V. , & Clarke, V. (2022). Thematic analysis: A practical guide. Sage.

[ajcp70042-bib-0006] Bryant‐Davis, T. , Ullman, S. E. , Tsong, Y. , & Gobin, R. (2011). Surviving the storm: The role of social support and religious coping in sexual assault recovery of African American women. Violence Against Women, 17(12), 1601–1618. 10.1177/1077801211436138 22410773 PMC3844285

[ajcp70042-bib-0007] Campbell, R. (2024). Systems‐centered care versus survivor‐centered care: Reimagining help and healing for sexual assault survivors. Psychology of Violence, 14(6), 379–385. 10.1037/vio0000528

[ajcp70042-bib-0008] Campbell, R. , Dworkin, E. , & Cabral, G. (2009). An ecological model of the impact of sexual assault on women's mental health. Trauma, violence & abuse, 10(3), 225–246. 10.1177/1524838009334456 19433406

[ajcp70042-bib-0009] Campbell, R. , & Feeney, H. (2023). “On the shelves, covered in dust:” The history of untested sexual assault kits (SAKs) in the United States. In R. Lovell , & J. Langhinrichsen‐Rohling (Eds.), Sexual Assault Kits and Reforming the Response to Rape (pp. 9–23). Routledge.

[ajcp70042-bib-0010] Campbell, R. , Fehler‐Cabral, G. , Pierce, S. J. , Sharma, D. B. , Shaw, J. , Horsford, S. , & Feeney, H. (2021). Changing the criminal justice system response to sexual assault: An empirical study of a participatory action research project. American Journal of Community Psychology, 67(1–2), 166–178. 10.1002/ajcp.12428 32511777

[ajcp70042-bib-0011] Campbell, R. , Goodman‐Williams, R. , Engleton, J. , Javorka, M. , & Gregory, K. (2023). Open science and data sharing in trauma research: Developing a trauma‐informed protocol for archiving sensitive qualitative data. Psychological Trauma: Theory, Research, Practice and Policy, 15(5), 819–828. 10.1037/tra0001358 36074633

[ajcp70042-bib-0012] Campbell, R. , Greeson, M. R. , Fehler‐Cabral, G. , & Kennedy, A. C. (2015). Pathways to help: Adolescent sexual assault victims' disclosure and help‐seeking experiences. Violence Against Women, 21(7), 824–847. 10.1177/1077801215584071 25933673

[ajcp70042-bib-0013] Campbell, R. , Gregory, K. , Engleton, J. , Javorka, M. , & Goodman‐Williams, R. (2025). This time it was different:” Creating a multidisciplinary, trauma‐informed, victim‐centered approach to sexual assault cold case investigations and prosecutions. Journal of Interpersonal Violence, 40(15–16), 3639–3662. 10.1177/08862605241284068 39323175

[ajcp70042-bib-0014] Campbell, R. , Gregory, K. , Goodman‐Williams, R. , Engleton, J. , & Javorka, M. (2023). Victim notification protocols for untested sexual assault kits: survivors' and advocates' perspectives on law enforcement‐led outreach methods. Violence Against Women, 29(15–16), 3101–3125. 10.1177/1077801223120047 37700717

[ajcp70042-bib-0015] Campbell, R. , Gregory, K. , Goodman‐Williams, R. , Engleton, J. , & Javorka, M. (2024). Community‐based advocacy in “cold case” sexual assault prosecutions: A qualitative exploration of survivors' and advocates' experiences. Qualitative Social Work, 23(1), 126–144. 10.1177/14733250231207344

[ajcp70042-bib-0016] Campbell, R. , Gregory, K. , Goodman‐Williams, R. , Javorka, M. , & Engleton, J. (2025). The prosecution of sexual assault cases with forensic DNA evidence: Survivors' testimony experiences. Psychology, Public Policy, & Law, 31(2), 204–216. 10.1037/law0000453

[ajcp70042-bib-0017] Campbell, R. , Gregory, K. , Javorka, M. , Engleton, J. , Goodman‐Williams, R. , & Fishwick, K. (2022). *Evaluating a victim notification protocols for untested sexual assault kits (SAKs)* (OVW Award 2018‐SI‐AX‐0001 Final Report). Washington, DC: Office on Violence Against Women. 10.1177/10778012231200479 37700717

[ajcp70042-bib-0018] Catabay, C. J. , Stockman, J. K. , Campbell, J. C. , & Tsuyuki, K. (2019). Perceived stress and mental health: The mediating roles of social support and resilience among black women exposed to sexual violence. Journal of Affective Disorders, 259, 143–149. 10.1016/j.jad.2019.08.037 31445340 PMC6791774

[ajcp70042-bib-0019] Crenshaw, K. (1991). Mapping the margins: Intersectionality, identity politics, and violence against women of color. Stanford Law Review, 43(6), 1241–1299.

[ajcp70042-bib-0020] Creswell, J. W. , & Poth, C. N. (2025). Qualitative inquiry and research design: Choosing among five approaches. Sage.

[ajcp70042-bib-0021] Dworkin, E. R. , Brill, C. D. , & Ullman, S. E. (2019). Social reactions to disclosure of interpersonal violence and psychopathology: A systematic review and meta‐analysis. Clinical Psychology Review, 72, 101750. 10.1016/j.cpr.2019.101750 31260816 PMC6716998

[ajcp70042-bib-0022] Edwards, K. M. , Turchik, J. A. , Dardis, C. M. , Reynolds, N. , & Gidycz, C. A. (2011). Rape myths: History, individual and institutional‐level presence, and implications for change. Sex Roles, 65(11–12), 761–773. 10.1007/s11199-011-9943-2

[ajcp70042-bib-0023] Fedina, L. , Bright, C. L. , Campbell, R. , Rosay, A. B. , & Edmondson Smith, M. (2020). Experiences of sexual assault, economic insecurity, and health in an ethnically diverse sample of women. Psychology of Violence, 10(4), 355–366. 10.1037/vio0000272

[ajcp70042-bib-0024] Gómez, J. M. (2023). The cultural betrayal of Black women and girls: A Black feminist approach to healing from sexual abuse. APA Books.

[ajcp70042-bib-0025] Hlavka, H. R. , & Mulla, S. (2021). Bodies in evidence: Race, gender, and science in sexual assault adjudication. NYU Press.

[ajcp70042-bib-0026] Javorka, M. , & Campbell, R. (2025). “I don't feel that morally I had a choice:” Untested sexual assault kits and sexual assault survivors’ decisions to re‐engage with the criminal legal system. Psychology of Women Quarterly, 49(1), 106–121. 10.1177/03616843241304770

[ajcp70042-bib-0027] Kelley, S. M. (2023). Post‐sexual assault decision making: Centering Black women's experiences. Feminist Criminology, 18(2), 133–155. 10.1177/15570851221150912

[ajcp70042-bib-0028] Konradi, A. (2007). Taking the stand: Rape survivors and the prosecution of rapists. Praeger.

[ajcp70042-bib-0029] Long, L. , & Ullman, S. E. (2016). Correlates of problem drinking and drug use in black sexual assault victims. Violence and Victims, 31(1), 71–84. 10.1891/0886-6708.VV-D-14-00024 26646054

[ajcp70042-bib-0030] Lorenz, K. , Kirkner, A. , & Ullman, S. E. (2019). A qualitative study of sexual assault survivors' post‐assault legal system experiences. Journal of Trauma & Dissociation: The Official Journal of the International Society for the Study of Dissociation (ISSD), 20(3), 263–287. 10.1080/15299732.2019.1592643 31072270 PMC6994185

[ajcp70042-bib-0031] Lovell , & Langhinrichsen‐Rohling, J. (Eds.) (2023). Sexual assault kits and reforming the response to rape. Routledge.

[ajcp70042-bib-0032] Lovell, R. E. , Fletcher, A. M. C. , Sabo, D. , Overman, L. , & Flannery, D. J. (2023). What an examination of previously untested sexual assault kits tells us about the patterns of victimization and case outcomes for Black women and girls. In E. M. Ahlin , O. Mitchell , & C. A. Atkin‐Plunk (Eds.), Handbook on inequalities in sentencing and corrections (pp. 49–69). Routledge.

[ajcp70042-bib-0033] McGuire, D. L. (2010). At the dark end of the street: Black women, rape and resistance—a new history of the civil rights movement from Rosa Parks to the rise of black power. Vintage.

[ajcp70042-bib-0034] Patterson, D. (2011a). The impact of detectives' manner of questioning on rape victims' disclosure. Violence Against Women, 17(11), 1349–1373. 10.1177/1077801211434725 22240403

[ajcp70042-bib-0035] Patterson, D. (2011b). The linkage between secondary victimization by law enforcement and rape case outcomes. Journal of Interpersonal Violence, 26(2), 328–347. 10.1177/0886260510362 20237390

[ajcp70042-bib-0036] Patterson, D. , & Campbell, R. (2010). Why rape survivors participate in the criminal justice system. Journal of Community Psychology, 38(2), 191–205. 10.1002/jcop.20359

[ajcp70042-bib-0037] Patton, M. Q. (2015). Qualitative research & evaluation methods: Integrating theory and practice. Sage.

[ajcp70042-bib-0038] Relyea, M. , & Ullman, S. E. (2015). Unsupported or turned against: Understanding how two types of negative social reactions to sexual assault relate to postassault outcomes. Psychology of Women Quarterly, 39(1), 37–52. 10.1177/0361684313512610 25750475 PMC4349407

[ajcp70042-bib-0039] Richie, B. E. , & Eife, E. (2021). Black bodies at the dangerous intersection of gender violence and mass criminalization. Journal of Aggression, Maltreatment & Trauma, 30(7), 877–888.

[ajcp70042-bib-0040] Sexual Assault Kit Initiative (SAKI) (2025). Retrieved from https://www.sakitta.org/

[ajcp70042-bib-0041] Shaw, J. , & Lee, H. (2019). Race and the criminal justice system response to sexual assault: A systematic review. American Journal of Community Psychology, 64(1–2), 256–278. 10.1002/ajcp.12334 PMC6796211 31059130

[ajcp70042-bib-0042] Slatton, B. C. , & Richard, A. L. (2020). Black women's experiences of sexual assault and disclosure: Insights from the margins. Sociology Compass, 14(6), e12792. 10.1111/soc4.12792

[ajcp70042-bib-0043] Spohn, C. (2020). Sexual assault case processing: The more things change, the more they stay the same. International Journal for Crime, Justice and Social Democracy, 9(1), 86–94. 10.5204/ijcjsd.v9i1.1454

[ajcp70042-bib-0044] Stockman, D. , Haney, L. , Uzieblo, K. , Littleton, H. , Keygnaert, I. , Lemmens, G. , & Verhofstadt, L. (2023). An ecological approach to understanding the impact of sexual violence: A systematic meta‐review. Frontiers in Psychology, 14, 1–16. 10.3389/fpsyg.2023.1032408 PMC10244654 37292501

[ajcp70042-bib-0045] Strom, K. , Scott, T. , Feeney, H. , Young, A. , Couzens, L. , & Berzofsky, M. (2021). How much justice is denied? An estimate of unsubmitted sexual assault kits in the United States. Journal of Criminal Justice, 73, 101746. 10.1016/j.jcrimjus.2020.101746

[ajcp70042-bib-0046] Ullman, S. E. (2023). Correlates of social reactions to victims' disclosures of sexual assault and intimate partner violence: A systematic review. Trauma, Violence & Abuse, 24(1), 29–43. 10.1177/15248380211016013 34008446

[ajcp70042-bib-0047] Ullman, S. E. (2024). Talking about sexual assault: Society's response to survivors (2nd ed.). APA Books.

[ajcp70042-bib-0048] Washington, P. A. (2001). Disclosure patterns of Black female sexual assault survivors. Violence Against Women, 7(11), 1254–1283. 10.1177/10778010122183856

[ajcp70042-bib-0049] Watson, L. B. , Robinson, D. , Dispenza, F. , & Nazari, N. (2012). African American women's sexual objectification experiences: A qualitative study. Psychology of Women Quarterly, 36(4), 458–475. 10.1177/0361684312454724

